# Feco-prevalence and risk factors of *Helicobacter pylori* infection among adults with dyspepsia in Mogadishu, Somalia: a cross-sectional study

**DOI:** 10.1186/s13099-026-00807-7

**Published:** 2026-02-09

**Authors:** Mohamed Yusuf Abdi, Rowdo Mohamed Mohamud, Salma Yusuf Abdullahi

**Affiliations:** Department of Microbiology and Laboratory Sciences, Faculty of Medicine and Health Sciences, SIMAD University, Mogadishu, Somalia

**Keywords:** Helicobacter pylori, Dyspepsia, Prevalence, Risk factors, Somalia

## Abstract

**Background:**

*Helicobacter pylori* is one of the most prevalent bacterial infections in humans, affecting more than half of the global population. It is a major contributor to gastric disorders among patients attending gastroenterology clinics. Therefore, this study aimed to evaluate the fecal prevalence and associated factors of *Helicobacter pylori* infection in adult patients with dyspepsia in Mogadishu, Somalia.

**Methods:**

A cross-sectional investigation was conducted at Shaafi Hospital between February and June 2025, encompassing 385 adult patients with dyspepsia. Researchers employed structured questionnaires to collect sociodemographic data and *Helicobacter pylori* infection-associated factors. *Helicobacter pylori* fecal antigen was detected in stool samples using lateral flow immunochromatographic assay. Binary and multiple logistic regression analyses were performed to identify the factors involved. A statistical association between variables was recognized when the p-value was below 0.05, ensuring a 95% confidence level.

**Results:**

Out of 385 adult patients with dyspepsia examined, 40% (*n* = 154) tested positive for *Helicobacter pylori* infection. Bivariate analysis revealed several factors linked to the infection: residing with children or elderly individuals (OR = 0.447, *p* = 0.014), consuming unwashed fruits or vegetables (OR = 1.658, *p* = 0.017), and eating white meat (OR = 1.986, *p* = 0.001). However, in the multivariate analysis, only two factors remained significant: living with children or elderly people was associated with a reduced likelihood of infection (AOR = 0.471, *p* = 0.015), whereas consumption of white meat was associated with a higher risk of infection (AOR = 1.699, *p* = 0.019). Other factors, such as hygiene habits, income, education, and smoking, did not show statistically significant associations.

**Conclusion:**

This study found a notable prevalence of *Helicobacter pylori* infection among adults with dyspepsia. Consumption of white meat was associated with an increased risk of infection, whereas living with children or elderly individuals appeared to provide some protection. These findings highlight the need for targeted public health strategies and suggest the need for further investigation into dietary and household factors that may influence *Helicobacter pylori* transmission.

## Background

Dyspepsia, often referred to as indigestion, is characterized by persistent or repeated discomfort in the upper abdomen, including symptoms such as pain, a sense of fullness, bloating, nausea, and feeling full quickly. It is generally divided into two primary types: organic dyspepsia, where a structural or biochemical issue, such as peptic ulcer disease (PUD), cancer, or gastrointestinal reflux; and functional dyspepsia (FD), formerly known as non-ulcer dyspepsia, where no organic cause is found even after comprehensive evaluation, including endoscopy. Functional dyspepsia is the most common subtype, with research indicating that more than 70% of people experiencing dyspeptic symptoms do not show abnormalities on endoscopy, thereby qualifying for FD diagnosis [1].

In a 2024 meta-analysis that utilized the Rome criteria across various adult populations globally, the estimated worldwide prevalence of functional dyspepsia was 8.4% (95% CI: 7.4–9.5%) [2]. Its prevalence was significantly higher in low- and middle-income countries (LMICs). A study conducted across multiple countries found an average rate of 37.9%, with figures ranging from approximately 26% to 44%. These rates are particularly high among Afro-Caribbean populations such as Sudan (44.3%) and Egypt (41.4%) [3]. In sub-Saharan Africa, data collected from communities in Rwanda indicated a dyspepsia prevalence rate of 14.2% when strict criteria were used. However, previous research focusing on specific populations, such as patients with diabetes in Kenya, found significantly higher rates, up to 53% [4]. When dyspepsia is not treated, it can lead to diminished quality of life for patients, manifesting in issues such as anxiety, depression, and somatization [5].

A recent review from Africa reported a *H. pylori* prevalence of approximately 79.1%, highlighting its widespread presence and its contribution to dyspepsia and peptic ulcer disease in the area [6]. Similarly, studies from other regions of the Arab world have revealed notable infection rates in the general population. For example, a study conducted in North Lebanon found a 31% prevalence among healthy volunteers [7], whereas another study in the United Arab Emirates reported a 41% prevalence among healthy, asymptomatic individuals [8].

In people with functional dyspepsia (FD), untreated *H. pylori* infection can lead to significant long-term complications, even in the absence of visible structural issues. The main risks involve the onset of peptic ulcer disease, chronic atrophic gastritis, and potentially gastric cancer, such as gastric adenocarcinoma and MALT lymphoma [9]. Additionally, the infection might cause hypochlorhydria, which impairs digestion and nutrient absorption, and could also lead to gastric motility disorders [10]. While not all FD patients face complications, the presence of *H. pylori* increases healthcare usage due to recurring symptoms and the necessity for diagnostic evaluations, underscoring the importance of early detection and treatment. Moreover, no study has been conducted on the feco-prevalence and related risk factors of *H. pylori* infection among adults with dyspepsia in Mogadishu, Somalia. Hence, the current study aimed to determine the prevalence of *H. pylori* infection in dyspeptic patients by using a stool antigen test, which is effective in detecting acute infections and evaluating potential risk factors.

## Methodology

### Study design, study area and study population

This cross-sectional study was conducted at the outpatient department of Shaafi Hospital, a private, multi-specialty healthcare facility situated in the Hodan district of Mogadishu, Banaadir region, from February 1st to April 30, 2025. The study included patients aged 18 years and older who were experiencing dyspepsia. Individuals who had been previously diagnosed with an active peptic ulcer or stomach cancer, as well as those who had undergone *H. pylori* eradication treatment in the past three months, were not included in the study.

### Sample size determination and sampling techniques

The formula for a single-population proportion was used to calculate the required sample size for estimating prevalence, based on a 50% prevalence rate, a 5% margin of error, and a 95% confidence interval. Consequently, the sample size was rounded up to include 385 participants. Study participants were selected using a simple random sampling technique.

### Data collection and variables

Data on the sociodemographic, environmental, and behavioral characteristics of the study participants were collected using a structured, pretested questionnaire administered through face-to-face interviews conducted in Somali. The principal investigator trained the data collectors on the data collection process. The participants were asked to provide a fecal sample weighing approximately 50 mg. This study primarily focused on the prevalence of *H. pylori* infection in adult dyspepsia patients. The independent variables included some of potential risk factors associated with *H. pylori* infection which could have influenced its prevalence in the study group. These factors encompassed a wide range of sociodemographic, behavioral, environmental, and lifestyle characteristics of the participants. The sociodemographic variables considered were gender, age group, education level, occupation, monthly income in USD, marital status, and family size. The behavioral factors examined included habits such as smoking or khat chewing, consumption of spicy or fatty foods, intake of caffeinated beverages (such as coffee, tea, and energy drinks), and the frequency of consumption of red meat, white meat, and fast food. Additionally, environmental and lifestyle-related risk factors were evaluated, including whether participants drank untreated or unfiltered water, kept domestic animals in their households, practiced handwashing before meals and after using the toilet, and emptied trash bags daily. These variables were analyzed to explore their potential impact on the prevalence of *H. pylori* infection in the target population.

### Specimen collection and laboratory analysis

Each dyspeptic patient was provided a clean, dry, wide-mouthed, screw-capped plastic container without disinfectants or preservatives. The participants were clearly instructed, in Somali language, on how to collect a fresh stool sample. Approximately 5 g of stool sample was collected from each patient. The samples were labeled with unique codes, time-stamped, and immediately placed in a cool box (2–8 °C) for transport to the Microbiology Lab, Faculty of Medicine and Health Sciences, SIMAD University. All the samples were immediately processed to ensure antigen stability. The samples were analyzed in the *H. pylori* Stool Antigen Rapid Test Kit (One-Step H. pylori Feces Test, Guangzhou Wondfo Biotech, China). The diagnostic test employs a lateral flow immunochromatographic assay, which achieves a sensitivity of 99.20% and specificity of 99.26% for the detection of *H. pylori* antigens in stool specimens [11].

### Data analysis

The data collection and analysis were conducted using the Statistical Package for Social Sciences (SPSS) version 27. Descriptive statistics were obtained for the demographic information. Categorical variables were presented as frequencies and proportions. Frequency analyses were performed to evaluate the occurrence of *H. pylori* infections. To identify the factors linked to *H. pylori* infection, both bivariate and multivariate analyses were utilized. Bivariate analysis involved calculating crude odds ratios and p-values through chi-square tests, with results shown in a table and statistical significance set at *p* < 0.05. Variables with a p-value < 0.20 were subsequently included in the multivariate logistic regression to determine adjusted associations. The outcomes of the multivariate analysis, which included adjusted odds ratios and p-values, are also displayed in the tables, maintaining a significance level of *p* < 0.05.

## Results

### Sociodemographic characteristics of the study participants

The demographic characteristics of dyspepsia patients revealed that the majority were male, accounting for 60.0%, and 80.8% were aged 45 years or younger. Most of these patients were married (62.9%), while 27.5% were single, and 9.6% were either widowed or divorced. In terms of educational background, 72.7% had received formal education, whereas 27.3% had informal education. Regarding employment, 56.1% were employed, and 43.9% were unemployed. When it came to monthly income, 53.0% earned over 300 USD, while 47.0% had an income of 300 USD or less. Furthermore, 66.5% of the patients belonged to families with more than five members, while 33.5% were from families with five or fewer members (Table 1).

**Table 1:** Sociodemographic characteristics of the study participants

Variable	F (%)
**Gender**	
Male	231 (60.0%)
Female	154 (40.0%)
**Age**	
≤ 45 years	311 (80.8%)
˃ 45 years	74 (19.2%)
**Marital status**	
Single	106 (27.5%)
Married	242 (62.9%)
Widowed /divorced	37 (9.6%)
**Income (USD/month)**	
≤ 300	181 (47.0%)
˃ 300	204 (53.0%)
**Education status**	
Informal education	105 (27.3%)
Formal education	280 (72.7%)
**Occupational status**	
Employed	216 (56.1%)
Un-employed	169 (43.9%)
**Family size**	
≤ 5	129 (33.5%)
˃ 5	256 (66.5%)

### Environmental and behavioral characteristics of the study participants’ households

The data highlight significant trends in household hygiene habits and living conditions. A large majority of respondents (96.9%) frequently reported eating meals prepared outside their homes. Furthermore, 91.7% of participants reported drinking untreated water. The results for hygiene practices were generally positive. A significant number of individuals consistently practiced hand hygiene, with 95.1% and 99.7% washing their hand meals and after using the toilet, respectively. Regarding household demographics, 86.5% of participants reported living with children or older adults. Additionally, 34.0% of respondents stated that they kept domesticated animals. Regarding housing conditions, 57.4% of respondents reported having more than four rooms. Finally, an overwhelming majority (98.4%) confirmed that household waste was disposed of daily (Table 2).

**Table 2:** Environmental and behavioral characteristics of the study participants’ households

Variable	F (%)
**Do you regularly consume meals prepared outside your household?**	
Yes	373 (96.9%)
No	12 (3.1%)
**Do you consume water that has not undergone any form of purification or treatment?**	
Yes	353 (91.7%)
No	32 (8.3%)
**Do you consistently practice hand hygiene before eating meals?**	
Yes	366 (95.1%)
No	19 (4.9%)
**Do you consistently wash your hands after using the toilet?**	
Yes	384 (99.7%)
No	1 (0.3%)
**Do you live in a house with children or elderly individuals?**	
Yes	333 (86.5%)
No	52 (13.5%)
**Are there any domesticated animals (e.g., pets or livestock) living in your household?**	
Yes	131 (34.0%)
No	254 (66.0%)
How many rooms are there in your household?	
≤ 4	164 (42.6%)
˃ 4	221 (57.4%)
**Is household waste disposed of on a daily basis?**	
Yes	379 (98.4%)
No	6 (1.6%)

### Dietary and lifestyle habits of the study participants

The data revealed distinct patterns of dietary behavior and substance use among the surveyed population. Most respondents (70.1%) did not consume spicy or heavily seasoned foods, suggesting a general preference for milder dietary options. Similarly, only 15.8% of the participants reported frequent consumption of high-fat foods. However, caffeine consumption was reported by more than half of the respondents (56.4%), indicating a moderate to high intake of caffeinated beverages such as coffee, tea, or energy drinks. A relatively high percentage (55.6%) of the individuals reported consuming fruits or vegetables without washing them first. In terms of protein sources, the data showed a balanced intake: 52.2% of respondents consumed white meat (e.g., poultry and fish), whereas 56.9% consumed red meat (e.g., beef and lamb). Only 1.6% of the participants reported smoking tobacco products, and an even smaller proportion (1.3%) reported chewing khat (Table 3).

**Table 3:** Dietary and lifestyle habits of the study participants

Variable	F (%)
**Do you regularly consume foods that are spicy or heavily seasoned?**	
Yes	115 (29.9%)
No	270 (70.1%)
**Do you frequently consume foods that are high in fat content?**	
Yes	61 (15.8%)
No	324 (84.2%)
**Do you regularly consume beverages that contain caffeine (e.g., coffee, tea, energy drinks)?**	
Yes	217 (56.4%)
No	168 (43.6%)
**Do you consume fruits or vegetables without washing them first?**	
Yes	214 (55.6%)
No	171 (44.4%)
Do you consume white meat such as poultry or fish as part of your diet?	
Yes	201 (52.2%)
No	184 (47.8%)
**Do you include red meat (e.g., beef, lamb) in your diet?**	
Yes	219 (56.9%)
No	166 (43.1%)
**Do you currently smoke tobacco products such as cigarettes?**	
Yes	6 (1.6%)
No	379 (98.4%)
**Do you engage in the chewing of khat?**	
Yes	5 (1.3%)
No	380 (98.7%)

### Prevalence of *Helicobacter pylori* antigen among dyspeptic patients

Out of the 385 stool samples tested, 154 (40%) tested positive for *H. pylori* antigen, while 231 (60%) tested negative.

### Bivariate and multivariate analysis of sociodemographic, environmental, dietary, and behavioral factors associated with *H. pylori* infection among adult dyspeptic patients

The bivariate analysis explored the relationships between a range of sociodemographic, environmental, and behavioral factors and the occurrence of *H. pylori* infection. This was done by calculating the crude odds ratios (ORs), 95% confidence intervals (CIs), and corresponding p-values. Although several variables exhibited differences in *H. pylori* positivity among the groups, only a few showed statistically significant links. Notably, residence in a household with children or elderly individuals was significantly associated with *H. pylori* positivity (OR: 0.477; 95% CI: 0.264–0.860; *p* = 0.014). Additionally, individuals who consumed unwashed fruits and vegetables (OR: 1.658; 95% CI: 1.093–2.515; *p* = 0.017) and white meat (OR: 1.986; 95% CI: 1.310–3.012; *p* = 0.001) had notably higher odds of infection. Conversely, factors such as gender, age, income, job status, and hygiene practices did not exhibit statistically significant associations (*p* > 0.05). Interestingly, smoking and khat chewing initially appeared to have associations with H. pylori positivity; however, owing to zero counts in some contingency cells, continuity corrections were applied to calculate the odds ratios. The resulting ORs (e.g., smoking: OR = 0.112; 95% CI: 0.006–2.010) suggested a possible inverse relationship, but the broad confidence intervals and small sample sizes limited the ability to draw conclusions (Table 4).

**Table 4:** Bivariate analysis of Sociodemographic, Environmental, Dietary, and behavioral factors associated with *H. pylori* infection among adult dyspeptic patients

Variables	F (%)	H. pylori screening		OR (95%CI)	*P*-value
		Positive *n* (%)	Negative *n* (%)		
**Gender**					
Male	231 (60.0%)	96 (41.6%)	135 (58.4%)	1.177 (0.775–1.788)	0.445
Female	154 (40.0%)	58 (37.7%)	96 (62.3%)	1	
**Age group**					
≤ 45 years	311 (80.8%)	120 (38.6%)	191 (61.4%)	0.739 (0.443–1.232)	0.246
> 45 years	74 (19.2%)	34 (45.9%)	40 (54.1%)	1	
**Educational status**					
Informal education	105 (27.3%)	34 (32.4%)	71 (67.6%)	0.638 (0.398–1.024)	0.063
Formal education	280 (72.7%)	120 (42.9%)	160 (57.1%)	1	
**Occupational Status**					
Employed	216 (56.1%)	90 (41.7%)	126 (58.3%)	0.853 (0.565–1.288)	0.451
Un-employed	169 (43.9%)	64 (37.9%)	105 (62.1%)	1	
**Marital status**					
Not Married	143 (37.1%)	58 (40.6%)	85 (59.4%)	1.038 (0.681–1.582)	0.863
Married	242 (62.9%)	96 (39.7%)	146 (60.3%)	1	
**Income (USD/month)**					
≤ 300	181 (47.0%)	72 (39.8%)	109 (60.2%)	0.983 (0.653–1.479)	0.934
˃ 300	204 (53.0%)	82 (40.2%)	122 (59.8%)	1	
**Family size**					
≤ 5	129 (33.5%)	54 (41.9%)	75 (58.1%)	1.123 (0.730–1.728)	0.597
˃ 5	256 (66.5%)	100 (39.1%)	156 (60.9%)	1	
**Do you regularly consume meals prepared outside your household?**					
Yes	373 (96.9%)	149 (39.9%)	224 (60.1%)	0.931 (0.290–2.989)	0.905
No	12 (3.1%)	5 (41.7%)	7 (58.3%)	1	
**Do you consume water that has not undergone any form of purification or treatment?**					
Yes	353 (91.7%)	144 (40.8%)	209 (59.2%)	1.516 (0.697–3.297)	0.294
No	32 (8.3%)	10 (31.2%)	22 (68.8%)	1	
**Do you consistently practice hand hygiene before eating meals?**					
Yes	366 (95.1%)	145 (39.6%)	221 (60.4%)	0.729 (0.289–1.838)	0.503
No	19 (4.9%)	9 (47.4%)	10 (52.6%)	1	
**Do you consistently wash your hands after using the toilet?**					
Yes	384 (99.7%)	153 (39.8%)	231 (60.2%)	-	1.00
No	1 (0.3%)	1 (100.0%)	0 (0.0%)	1	
**Do you live in a house with children or elderly individuals?**					
Yes	333 (86.5%)	125 (37.5%)	208 (62.5%)	0.477 (0.264–0.860)	**0.014**
No	52 (13.5%)	29 (55.8%)	23 (44.2%)	1	
**Are there any domesticated animals (e.g.**,** pets or livestock) living in your household?**					
Yes	131 (34.0%)	56 (42.7%)	75 (57.3%)	1.189 (0.774–1.824)	0.429
No	254 (66.0%)	98 (38.6%)	156 (61.4%)	1	
**How many rooms are there in your household?**					
≤ 4	164 (42.6%)	57 (34.8%)	107 (65.2%)	0.681 (0.449–1.033)	0.071
˃ 4	221 (57.4%)	97 (43.9%)	124 (56.1%)	1	
**Is household waste disposed of on a daily basis?**					
Yes	379 (98.4%)	152 (40.1%)	227 (59.9%)	1.339 (0.242–7.403)	0.738
No	6 (1.6%)	2 (33.3%)	4 (66.7%)	1	
**Do you regularly consume foods that are spicy or heavily seasoned?**					
Yes	115 (29.9%)	42 (36.5%)	73 (63.5%)	0.812 (0.517–1.273)	0.364
No	270 (70.1%)	112 (41.5%)	158 (58.5%)	1	
**Do you frequently consume foods that are high in fat content?**					
Yes	61 (15.8%)	27 (44.3%)	34 (55.7%)	1.232 (0.709–2.140)	0.459
No	324 (84.2%)	127 (39.2%)	197 (60.8%)	1	
**Do you regularly consume beverages that contain caffeine (e.g.**,** coffee**,** tea**,** energy drinks)?**					
Yes	217 (56.4%)	79 (36.4%)	138 (63.6%)	0.710 (0.471–1.071)	0.102
No	168 (43.6%)	75 (44.6%)	93 (55.4%)	1	
**Do you consume fruits or vegetables without washing them first?**					
Yes	214 (55.6%)	97 (45.3%)	117 (54.7%)	1.658 (1.093–2.515)	**0.017**
No	171 (44.4%)	57 (33.3%)	114 (66.7%)	1	
**Do you consume white meat such as poultry or fish as part of your diet?**					
Yes	201 (52.2%)	96 (47.8%)	105 (52.2%)	1.986 (1.310–3.012)	**0.001**
No	184 (47.8%)	58 (31.5%)	126 (68.5%)	1	
**Do you include red meat (e.g.**,** beef**,** lamb) in your diet?**					
Yes	219 (56.9%)	91 (41.6%)	128 (58.4%)	1.162 (0.769–1.757)	0.475
No	166 (43.1%)	63 (38.0%)	103 (62.0%)	1	
**Do you currently smoke tobacco products such as cigarettes?**					
Yes	6 (1.6%)	0 (0.0%)	6 (100.0%)	0.112 (0.006–2.010) *	0.117
No	379 (98.4%)	154 (40.6%)	225 (59.4%)	1	
**Do you engage in the chewing of khat?**					
Yes	5 (1.3%)	0 (0.0%)	5 (100.0%)	0.133 (0.007–2.43) *	0.148
No	380 (98.7%)	154 (40.5%)	226 (59.5%)	1	

*Because there were zero counts in the ‘Yes’ category for both smoking and khat chewing, it was not possible to reliably calculate the crude odds ratios. To facilitate computation, a continuity correction of 0.5 was used and Fisher’s exact test were employed for statistical analysis

p-values in bold indicate statistical significance (*p* < 0.05)

In the multivariate logistic regression analysis, two factors remained significantly linked to *H. pylori* positivity. Individuals living with children or elderly people had notably lower chances of infection (AOR = 0.471, 95% CI: 0.257**–**0.863, *p* = 0.015). Conversely, those who regularly consumed white meat had a higher odd of infection (AOR = 1.699, 95% CI: 1.090–2.647, *p* = 0.019). Other factors, including educational status, number of rooms, and fruit washing practices, were not statistically significant. Smoking and khat chewing presented estimation challenges owing to limited data, rendering them uninterpretable in the model (Table 5).

**Table 5:** Multivariate logistic regression analysis of factors independently associated with *H. pylori* infection among adult dyspeptic patients

Variables	H. pylori screening		AOR (95%CI)	*P*-value
	Positive *n* (%)	Negative *n* (%)		
**Educational status**				
Informal education	34 (32.4%)	71 (67.6%)	0.728 (0.423–1.254)	0.253
Formal education	120 (42.9%)	160 (57.1%)	1	
**How many rooms are there in your household?**				
≤ 4	57 (34.8%)	107 (65.2%)	0.721 (0.467–1.111)	0.138
˃4	97 (43.9%)	124 (56.1%)	1	
**Do you live in a house with children or elderly individuals?**				
Yes	125 (37.5%)	208 (62.5%)	0.471 (0.257–0.863)	**0.015**
No	29 (55.8%)	23 (44.2%)	1	
**Do you consume fruits or vegetables without washing them first?**				
Yes	97 (45.3%)	117 (54.7%)	1.195 (0.731–1.954)	0.477
N*o*	57 (33.3%)	114 (66.7%)	1	
**Do you consume white meat such as poultry or fish as part of your diet?**				
Yes	96 (47.8%)	105 (52.2%)	1.699 (1.090–2.647)	**0.019**
No	58 (31.5%)	126 (68.5%)	1	
**Do you regularly consume beverages that contain caffeine (e.g.**,** coffee**,** tea**,** energy drinks)?**				
Yes	79 (36.4%)	138 (63.6%)	0.842 (0.537–1.322)	0.456
No	75 (44.6%)	93 (55.4%)	1	
**Do you currently smoke tobacco products such as cigarettes?**				
Yes	0 (0.0%)	6 (100.0%)	-	1.00
No	154 (40.6%)	225 (59.4%)	1	
**Do you engage in the chewin*g*** **of khat*?***				
Yes	0 (0.0%)	5 (100.0%)	*-*	1.00
No	154 (40.5%)	226 (59.5%)	1	

p-values in bold indicate statistical significance (*p* < 0.05)

## Discussion

This study aimed to assess the prevalence of *H. pylori* among dyspeptic patients visiting Shaafi Hospital in Mogadishu, Somalia and to identify related sociodemographic, behavioral, and environmental risk factors. This study revealed an overall prevalence of 40%, highlighting a considerable infection burden among adults with dyspepsia.

This result is in agreement with the findings of another study conducted in Mizan Aman.

Town, Southwest Ethiopia (42.8%) [12] and Dar es Salaam, Tanzania (43.77%) [13]. Nevertheless, this figure is lower than the findings from other studies conducted in Eastern Uganda (48%) [14], Hawassa City, Ethiopia (48.5%) [15], Hossana City, Southwest Ethiopia (49.2%) [16], Yilmana Densa District, Northwest Ethiopia (51.1%) [17], Hosanna town, Southeast Ethiopia (51.4%) [18], Egypt (53.1%) [19], Baghdad, Iraq (63.8%) [20], and Douala metropolis City, Cameroon (64.39%) [21]. The incidence in our study was higher than that reported in studies conducted in Oromia, Ethiopia (23.0%) [22]; Arba Minch city, Southern Ethiopia (32.2%) [23]; Kathmandu, Nepal (32.9%) [24]; Debre Tabor, Ethiopia (34%) [25]; Mymensingh, North-Central Bangladesh (36%) [26]; and Gondar, Northwest Ethiopia (37.6%) [27]. The differences in *H. pylori* prevalence observed in various studies might be shaped by a complex combination of environmental factors, socioeconomic conditions, diagnostic methods, population health behaviors, variations in host immunity, dietary habits, or study-specific elements such as sample size.

This study explored the sociodemographic and behavioral factors influencing *H. pylori* infection and identified several important elements through both bivariate and multivariate analyses. Although some relationships changed after adjusting for confounding variables, two factors, household composition (living with children or elderly people) and the consumption of white meat, remained significant predictors of *H. pylori* infection. These results were examined in relation to similar studies conducted in Ethiopia and other nations.

In the multivariate analysis, people residing with children or elderly individuals had a significantly decreased likelihood of *H. pylori* infection (AOR = 0.471; 95% CI: 0.257–0.863; *p* = 0.015). This finding was consistent with the bivariate analysis, where the same factor was associated with a decreased likelihood of infection (OR = 0.447; 95% CI: 0.264–0.860; *p* = 0.013), suggesting a potentially protective role of household composition. However, contrasting observations have been reported in other Ethiopian regions. For example, research conducted at the Debre Tabor Comprehensive Specialized Hospital revealed that individuals from households with four or more children had a significantly higher chance of infection (AOR = 7.5; 95% CI: 1.7–33.6; *p* = 0.008), supporting the idea that crowded living conditions may promote oral or fecal-oral transmission of *H. pylori* through shared environments and objects [25].

Our study indicated that individuals who frequently consume white meat, such as poultry and fish, exhibit a significantly higher likelihood of *H. pylori* infection (AOR = 1.699; 95% CI: 1.090–2.647; *p* = 0.019). This correlation is consistent with the results of a study in Pune, India, which also found that fish consumption was significantly associated with a higher chance of *H. pylori* infection (OR = 2.35; *p* < 0.05) [28]. This suggests a recurring pattern in certain environments where dietary practices may play a role in the spread of infection. Additionally, the link between these factors might be partly attributed to contamination of raw white meat, especially poultry. Numerous studies have supported the notion of *H. pylori* contamination of white meat. For example, a surveillance study in Egypt detected *H. pylori* in chicken meat samples sold in retail shops [29]. Similarly, a study in Iran reported contamination of raw poultry meat samples, including chickens [30]. Conversely, a recent Brazilian study found no significant association between the consumption of white meat or fish and *H. pylori* infection [31]. These diverse results highlight the significance of context interpretation. In environments with limited resources or high population density, poor food handling, insufficient cooking methods, and restricted refrigeration can increase the likelihood of *H. pylori* spreading through contaminated food such as white meat.

Our bivariate analysis showed a significant association between the consumption of unwashed fruits and vegetables and *H. pylori* infection (OR = 1.658; 95% CI: 1.093–2.515; *p* = 0.017), supporting the fecal–oral transmission route. These results are consistent with those of previous studies. For example, studies conducted in Iran, Ethiopia and Egypt found that unwashed vegetables were significantly associated with *H. pylori* contamination and infection, respectively (*p* < 0.05) [32–34]. However, in our multivariate analysis, this association was not significant, possibly due to confounding factors such as access to clean water or general hygiene practices. This is consistent with the results of a study conducted in Ethiopia, which found no significant association between the intake of fruits and vegetables and the detection of *H. pylori* stool antigen (*p* > 0.05) [35]. The variation in research findings may stem from differences in diagnostic techniques, population traits, environmental cleanliness, and sample sizes. Furthermore, differences in food hygiene practices, local contamination levels, and reliance on self-reported information may have influenced the results.

In the current study, dietary habits, such as consumption of spicy or fatty foods, intake of caffeinated drinks, and the frequency of consuming red meat and fast food, were not significantly associated with *H. pylori* infection in either bivariate or multivariate analyses. These findings are consistent with several Ethiopian studies conducted in Assosa, Dessie, Adama, Arba Minch, Yilmana Densa, and Southwest Ethiopia, all of which found no significant association between the consumption of coffee or spicy foods and *H. pylori* infection [17, 18, 22, 23, 35, 36]. Similar conclusions have been drawn from research in Iraq and Bahrain, indicating that these dietary practices are unlikely to be significant risk factors for *H. pylori* infection [20, 37].

In this study, there was no significant link between hand hygiene habits, drinking water sources and *H. pylori* infection. This finding differs from those of several Ethiopian studies that identified poor hand hygiene and the use of unprotected water sources as notable risk factors. For instance, research conducted in Assosa [36], and Arba Minch [23] demonstrated that washing hands after using the toilet significantly reduced the risk of infection. Similarly, water sources were significantly associated with increased infection risk in Yilmana Densa and Adama Woreda [17, 22]. Conversely, research conducted in Gondar [27] and Arba Minch [23] found no notable correlations, which is consistent with our results. These varied outcomes imply that local elements such as sanitation systems, water quality, and behavioral habits probably affect how hygiene and water sources contribute to the transmission of *H. pylori*.

The present study did not find a significant association between domestic animals and *H. pylori* infection. This finding differs from the results in Mizan Aman Town, Southwest Ethiopia, where domestic animals were found to significantly increase the likelihood of infection (AOR = 13.33; 95% CI: 2.203–80.692; *p* < 0.05) [12]. This discrepancy might be attributed to differences in environmental factors, the nature of human-animal interactions, or the methodologies used in the studies.

Despite evaluating household factors such as the number of rooms and the habit of daily waste disposal, no statistically significant association with *H. pylori* infection was found in either bivariate or multivariate analyses. This suggests that these environmental elements may have a limited influence on the transmission of infection within this population. These findings align with those of a study conducted in Bahrain, which also found no significant association between the number of rooms in a household and *H. pylori* infection [37].

In this study, no significant association were found between *H. pylori* infection and any sociodemographic factors such as gender, age, education, occupation, marital status, income, or family size. This is consistent with results from the University of Gondar Hospital, Ethiopia [27], Debre Tabor Comprehensive Specialized Hospital, Ethiopia [25], and Yilmana Densa District, North West Ethiopia [17], where most sociodemographic variables did not show significant associations. However, some studies have reported different outcomes. For instance, in Assosa General Hospital, West Ethiopia, being female, living in rural areas, and having a low income were significantly related to *H. Pylori* infection [36], whereas in the University of Gondar Hospital, Ethiopia, only sex (male) was significantly associated with *H. pylori* infection in this study [27]. Studies from Dessie Referral Hospital, Ethiopia [35], and Mizan Aman Town, Southwest, Ethiopia [12], found that larger family size was associated with infection, while lower educational status was significant in Arba Minch General Hospital in Southern Ethiopia [23], Adama Woreda, Oromia, Ethiopia [22], and Bahrain [37]. These variations underscore the context-dependent nature of the sociodemographic influences on *H. pylori* infection.

### Limitations of the study

This study has several limitations. As it employed a cross-sectional design, it restricted the ability to determine causal relationships between risk factors and *H. pylori* infection. This study was conducted at a single private hospital in Mogadishu, which might not reflect the wider population, potentially affecting the generalizability of the results. Furthermore, certain variables, such as smoking and khat chewing, were infrequently observed, diminishing the power to identify significant associations.

## Conclusions

This study found a 40% prevalence of *H. pylori* infection among dyspeptic patients in Mogadishu, which is consistent with regional data. Notable risk factors included household composition, where living with children or the elderly reduced the risk of infection, and white meat consumption, which increased the risk, possibly due to contamination. Other factors, such as fruit and vegetable hygiene, handwashing, water source, and sociodemographic variables, showed no significant associations after adjustment. These findings emphasize the multifactorial and context-specific nature of *H. pylori* transmission and highlight the need for targeted interventions and additional research.

## Data Availability

The data supporting the findings of this study can be obtained from the corresponding author upon request.

## Abbreviations

H. pylori: Helicobacter pylori
PUD: Peptic ulcer disease
FD: Functional dyspepsia
LMICs: Low- and middle-income countries
MALT: Mucosa-associated lymphoid tissue
USD: United states dollar
SPSS: Statistical Package for Social Sciences
